# Molecular Markers of Dental Pulp Tissue during Orthodontic Tooth Movement: A Pilot Study

**DOI:** 10.1100/2012/236427

**Published:** 2012-04-30

**Authors:** Rohaya Megat Abdul Wahab, Shahrul Hisham Zainal Ariffin, Wong Woan Yeen, Nurul Atikah Ahmad, Sahidan Senafi

**Affiliations:** ^1^Department of Orthodontics, Faculty of Dentistry, Universiti Kebangsaan Malaysia, 50300 Kuala Lumpur, Malaysia; ^2^School of Biosciences and Biotechnology, Faculty of Science and Technology, Universiti Kebangsaan Malaysia, 43600 Bangi, Malaysia

## Abstract

Three specific orthodontic tooth movement genes, that is, *FCRL1*, *HSPG2*, and *LAMB2* were detected at upper first premolar (with appliance) dental pulp tissue by using GeneFishing technique as compared to lower first premolar (without appliance). These three differentially expressed genes have the potential as molecular markers during orthodontic tooth movement by looking at molecular changes of pulp tissue.

## 1. Introduction

Orthodontic tooth movement (OTM) is a biologic event which is facilitated by remodeling of periodontal ligament (PDL) and alveolar bone in response to the applied mechanical stimuli. The effects of orthodontic forces on the dental pulp showed histological changes mostly in pulpal blood flow and vascular tissue pressure [1]. According to Barwick and Ramsay [2], the orthodontic treatment had caused several effects on the dental pulp such as alteration in pulpal respiration rate, pulpal obliteration by secondary dentin formation, internal root resorption, and pulpal necrosis. All of these changes on the dental pulp caused by orthodontic forces had been well described through histological studies, but not much information had been reported on pulp changes during orthodontic treatment at the molecular and cellular levels. The objective of this study was to determine differentially expressed genes (DEGs) during orthodontic tooth movement (OTM).

## 2. Materials and Methods

Teeth samples were taken from an adolescent female patient aged 14 years old who had an orthodontic preadjusted appliance (bracket slot of 0.022′′  × 0.028′′) bonded to the labial surface of upper arch from the Department of Orthodontics, Faculty of Dentistry, Universiti Kebangsaan Malaysia, Kuala Lumpur. The upper right first premolar tooth was extracted on the 14th day and followed by extraction of the upper left first premolar tooth on the 28th day of treatment. The lower first premolar teeth without orthodontic appliance were extracted and designated as the control. Patient's informed consent was taken following the approval of the Research and Ethical Committee, Faculty of Dentistry, Universiti Kebangsaan Malaysia for the uses of human samples. The extracted teeth were grooved vertically from the centre of mesial and distal marginal ridge until cementoenamel junction using dental fissure burs without exposing the pulp chamber and cut into two-halves using a dental cutter. The pulp tissue was extracted using a barbed broach (size 10).

RNAs was extracted using Trizol Reagent (Invitrogen) following the manufacturer's instruction. GeneFishing technique was used to determine the differentially expressed genes (DEG) using the GeneFishing DEG kit (Arbitrary ACP 1–20) from SeeGene, Seoul, South Korea. It uses primers which anneal specifically to the template and allows genuine products to be amplified to reduce false-positive amplification. The amplified PCR products were separated using 2% (w/v) agarose gel and stained with ethidium bromide. Differentially expressed PCR products were extracted from the agarose gel using the Wizard SV Gel and PCR Clean-Up kit (Promega, Madison, WI, USA). Each DNA fragment was cloned using TOPO TA Cloning kit (Invitrogen, CA, USA). The recombinant DNA plasmids were extracted using Wizard Plus SV Minipreps DNA Purification System (Promega, Madison, USA). Sequence identity was confirmed by nucleotide BLAST (BLASTN) searches on the combined GenBank/EMBL reference mRNA sequences (refSeq_RNA), accessed through the National Center for Biotechnology Information homepage (http://www.ncbi.nlm.nih.gov/). Further information and pathways analysis of the genes obtained were gathered through the European Bioinformatics Institute homepage (http://www.ebi.ac.uk/), KEGG pathway database homepage (http://www.genome.jp/kegg/pathway.html), and Reactome database (http://www.reactome.org/ReactomeGWT/entrypoint.html).

## 3. Results and Discussions

**Figure 1:**
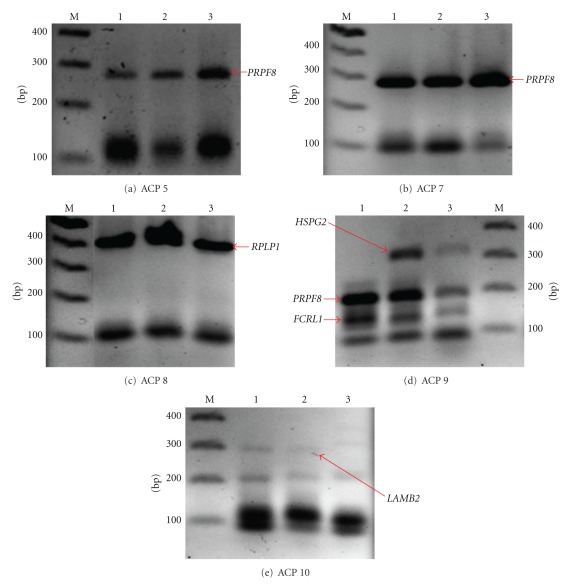
GeneFishing DEG screening results. There are 5 genes activated in pulp tissue (arrows). Lane 1: Normal; Lane 2: 14 days after force application; Lane 3: 28 days after force application; Lane M: 100 bp DNA marker.

The mRNA expression profiles of extracted teeth on the 14th and 28th days after orthodontic treatment were compared with normal pulp tissue to identify genes that specifically or predominantly expressed in pulp tissue during OTM. The mRNA from both types of tissues was extracted and subjected to ACP 1-20 RT-PCR analysis. The expression pattern of differentially expressed transcripts was obtained only when subjected to ACPs 5, 7, 8, 9, and 10 in response to the orthodontic treatment of various periods, that is, days 0, 14, and 28 as shown in Table 1 and Figure 1. All of the DEGs obtained were cloned, sequenced, and confirmed with GenBank. The sequence similarities of differentially expressed transcripts were analyzed using BLASTN analysis (http://www.ncbi.nlm.nih.gov/). ACPs 5, 7, and 8 which were used in this study, encoded the same gene expression before and after treatment except for ACPs 9 and 10 (Table 1; Figure 1).

The results of this study showed the expression of five specific genes, that is, *PRPF8, RPLP1*, *FCRL1*, *HSPG2,* and *LAMB2.* DNA amplifications of ACPs 5, 7, and 9 produced *PRPF8*, while amplification of ACP 8 yielded *RPLP1*. The expression pattern showed that *PRPF8 *and *RPLP1* genes were continuously activated before and after treatment. PCR amplification of ACP 9 produced *FCRL1* and *HSPG2*, while ACP 10 produced *LAMB2*. *FCRL1* gene was active at day 0 followed by lower activation at day 14 and slightly activated at day 28 of treatment. *HSPG2* gene was only activated at 14 days of treatment, and activation became lower at day 28. Our result demonstrated that at day 0,* LAMB2* gene was active and downregulated at day 14 and suppressed at day 28 of treatment (Table 1).

*PRPF8* and *RPLP1 *are housekeeping genes as identified by Eisenberg and Levanon [3]. Our study further proven that both of these genes are housekeeping genes because they were actively expressed in dental pulp tissues before and after orthodontic treatment. (Table 1; Figures 1(a), 1(b), 1(c), and 1(d)). *PRPF8 *encodes U5 snRP-specific protein which is essential in RNA and mRNA splicing through transesterification [4] and spliceosome [5] processes, respectively. Meanwhile, *RPLP1* gene encodes 60S acidic ribosomal protein P1. This protein plays an important role in the elongation step of protein synthesis and is also involved in pathway, where it functions mostly as translational control [6].

Fc receptor-like 1 gene (*FCRL1*) was detected in this study by the amplification of ACP 9. The FCRL1 transmembrane glycoprotein encoded by *FCRL1 *gene, is one of the immunoglobulin Fc receptor homolog (FcRH) expressed by human B cell. FCRL1 which potentially serve as an activating coreceptor in B cells begin to be expressed in pre-B cells, reaching peak levels on naive B cells (inactive B cell) and is downregulated after B cells are activated to begin the formation of germinal center [7]. In our study, *FCRL1* gene was found to be active at day 0 when there was no application of mechanical stress, hence suggesting that inactive B cells were found in dental pulp tissue before OTM. At day 14, the *FCRL1* gene was still active, but the activation was lower compared to control. This could be due to the start of activation of B cell. At day 28, the *FCRL1* gene was downregulated even more which probably because B cells were actively induced and inflammation occurred as also shown by Rohaya et al. [8] using aspartate aminotransferase as biomarker.

B cells are not only involved in adaptive immune system but also participated in bone homeostasis during normal physiology [9]. During normal physiology, mature B cell produces >50% of total bone-marrow-derived osteoprotegerin in order to restrain osteoclastogenesis [10]. Therefore, the activation of *FCRL1* gene at day 0 indicated the presence of naive B cells (inactive B cell) that were needed to restrain osteoclastogenesis. Following 14 and 28 days of treatment, *FCRL* gene was gradually decreased probably due to the activation of B cell to start osteoclastogenesis. Our finding was in concordance with the study from Rohaya et al. [11] which showed that bone resorption occurred at weeks 3 and 4 using tartrate resistant acid phosphatase as the indicator.

In our study, heparan sulfate proteoglycan 2 gene (*HSPG2*) was activated when amplified by ACP 9 at day 14 after orthodontic force was applied (Table 1; Figure 1(d)). *HSPG2* gene encodes a protein named perlecan (PLC). PLC is an integral component of basement membranes which serves as an attachment substrate for cells. It plays an important role during cell adhesion, chondrocyte differentiation, endochondral ossification, extracellular matrix (ECM) organization as well as cartilage development involved in endochondral bone morphogenesis process [12]. PLC is one of the major molecules in the ECM which contribute to withstand the mechanical stress and is necessary in the remodeling of the tissue [13]. At day 0, there was no activation of *HSPG2* gene as the pulp tissue was in normal condition. Some studies reported that mechanical strain stimulated the production of PLC and heparin sulphate glycosaminoglycan by endothelial cells [14]. Similarly, our result demonstrated that PLC was expressed when mechanical forces were applied at day 14. This suggested that PLC is important in repairing and remodeling ECM in tissue stroma and basement membrane. Expression of PLC was downregulated on the 28th day of treatment. This could be due to less archwire deflection as the teeth on the upper arch had begun to align, thus reducing the mechanical stress. However, further research is needed to conclude the involvement of PLC during orthodontic applied force.

Laminin, beta 2 protein (LAMB2) or also known as laminin S (LAMS) is encoded by *LAMB2 *gene. LAMB2 protein is mostly secreted in extracellular space, ECM, and basement membrane and is implicated in mediating the attachment, migration, and organization of cells into tissues by interacting with other ECM components. The interaction between ECM and cells is assisted by the binding of high-affinity receptors (integrins), and this interaction leads to a pathway known as integrin cell surface pathway [15]. In normal condition, *LAMB2 *gene was active as there was no mechanical stress to disturb the organization of the ECM and cells. According to the study by Gersdorff et al. [16], gene expression of laminin alpha2, alpha4, beta 1, beta 2, and gamma 3 chains was significantly downregulated in inflamed PDL cells. Our result indicated that, at day 14 of orthodontic treatment which is often associated with inflammation, *LAMB2* gene was downregulated and eventually suppressed at day 28. It is proposed that the suppression rate of *LAMB2* gene was slow due to the insensitivity response towards the mechanical force. However, further research is definitely needed to investigate the response of this gene to mechanical forces and inflammation during OTM.

## 4. Conclusion

This study showed that the three specific genes, that is, *FCRL1*, *HSPG2,* and *LAMB2 *that were detected during orthodontic treatment were found to be important during OTM. These differentially expressed genes could act as potential molecular markers to monitor the progression of orthodontic treatment.

## Figures and Tables

**Table 1:** Activation pattern of specific genes in response to orthodontic treatment at various periods.

Gene	ACP	Activation time (day)		
		0	14	28
	5	+	+	+
*PRPF8*	7	+	+	+
	9	+	+	+
*RPLP1*	8	+	+	+
*FCRL1*	9	+	+	Low +
*HSPG2*	9	−	+	Low +
*LAMB2*	10	+	Low +	−

*PRPF8*:* Homo sapiens* PRP8 pre-mRNA processing factor 8 homolog (*S.cerevisiae*) [NM_006445], *RPLP1*: *Homo sapiens* ribosomal protein, large, P1, transcript variant 1 [NM_001003], *FCRL1*: *Homo sapiens* Fc receptor-like 1, transcript variant 3 [NM_001159398], *HSPG2*: *Homo sapiens *heparan sulfate proteoglycan 2 [NM_005529], *LAMB2*: *Homo sapiens *laminin beta 2 (laminin S) [NM_002292], +: genes were activated, low +: genes were low-activated, and −: genes were not activated or suppressed.

## References

[1] Cohen S, Hagreaves KM (2006). *Pathways of the Pulp*.

[2] Barwick PJ, Ramsay DS (1996). Effect of brief intrusive force on human pulpal blood flow. *American Journal of Orthodontics and Dentofacial Orthopedics*.

[3] Eisenberg E, Levanon EY (2003). Human housekeeping genes are compact. *Trends in Genetics*.

[4] Achsel T, Ahrens K, Brahms H, Teigelkamp S, Lührmann R (1998). The human U5-220kD protein (hPrp8) forms a stable RNA-free complex with several U5-specific proteins, including an RNA unwindase, a homologue of ribosomal elongation factor EF-2, and a novel WD-40 protein. *Molecular and Cellular Biology*.

[5] Zhou Z, Licklider LJ, Gygi SP, Reed R (2002). Comprehensive proteomic analysis of the human spliceosome. *Nature*.

[6] Gebauer F, Hentze MW (2004). Molecular mechanisms of translational control. *Nature Reviews Molecular Cell Biology*.

[7] Leu CM, Davis RS, Gartland LA, Fine WD, Cooper MD (2005). FcRH1: an activation coreceptor on human B cells. *Blood*.

[8] Rohaya MAW, Shahrul Hisham ZA, Khazlina K (2009). Preliminary study of aspartate aminotransferase activity in gingival crevicular fluids during orthodontic tooth movement. *Journal of Applied Sciences*.

[9] Li Y, Toraldo G, Li A (2007). B cells and T cells are critical for the preservation of bone homeostasis and attainment of peak bone mass in vivo. *Blood*.

[10] Raggatt LJ, Partridge NC (2010). Cellular and molecular mechanisms of bone remodeling. *Journal of Biological Chemistry*.

[11] Rohaya MAW, Dasor MM, Senafi S (2011). Crevicular tartrate resistant acid phosphatase activity and rate of tooth movement under different continuous force application. *African Journal of Pharmacy and Pharmacology*.

[12] Mongiat M, Fu J, Oldershaw R, Greenhalgh R, Gown AM, Iozzo RV (2003). Perlecan protein core interacts with extracellular matrix protein 1 (ECM1), a glycoprotein involved in bone formation and angiogenesis. *Journal of Biological Chemistry*.

[13] Echtermeyer F, Baciu PC, Saoncella S, Ge Y, Goetinck PF (1999). Syndecan-4 core protein is sufficient for the assembly of focal adhesions and actin stress fibers. *Journal of Cell Science*.

[14] Baker AB, Ettenson DS, Jonas M, Nugent MA, Iozzo RV, Edelman ER (2008). Endothelial cells provide feedback control for vascular remodeling through a mechanosensitive autocrine TGF-*β* signaling pathway. *Circulation Research*.

[15] Faull RJ, Ginsberg MH (1996). Inside-out signaling through integrins. *Journal of the American Society of Nephrology*.

[16] Gersdorff N, Miró X, Roediger M (2008). Gene expression analysis of chronically inflamed and healthy human periodontal ligament cells in vivo. *Dental Research Journal*.

